# Plasticity drives extreme cold tolerance of emerald ash borer (*Agrilus planipennis*) during a polar vortex

**DOI:** 10.1016/j.cris.2022.100031

**Published:** 2022-02-03

**Authors:** Meghan E. Duell, Meghan T. Gray, Amanda D. Roe, Chris J.K. MacQuarrie, Brent J. Sinclair

**Affiliations:** 1 Department of Biology, University of Western Ontario, London, Ontario, Canada; 2 Great Lakes Forestry Centre, Canadian Forest Service, Natrual Resources Canada, Sault Ste. Marie, Ontario, Canada

**Keywords:** Invasive species, Forest pests, Overwintering, Freeze avoidance, Glycerol, Acclimatisation

## Abstract

Invasive species must often survive combinations of environmental conditions that differ considerably from their native range; however, for a given species it is unclear whether improved tolerance is the result of phenotypic plasticity or genetic adaptation (or both). *Agrilus planipennis* (Coleoptera: Buprestidae; the emerald ash borer) is an invasive pest of *Fraxinus* trees in North America and Europe. Previous studies in SW Ontario, Canada, showed that *A. planipennis* is freeze avoidant, preventing internal ice formation by accumulating Molar concentrations of glycerol in its hemolymph and depressing its supercooling point (SCP, the temperature at which it freezes). The cold tolerance of these SW Ontario animals was used to predict potential distribution, revealing that some Canadian cities should be too cold to allow populations to persist. However, a small population of *A. planipennis* has persisted in Winnipeg, Manitoba, Canada, through several severe ‘polar vortex’ events. In 2018/19, we collected *A. planipennis* larvae and prepupae from Winnipeg, MB and Southern Ontario, and found that individuals from Winnipeg were extremely cold tolerant – with SCPs as low as -52°C in prepupae (compared to -32°C in SW Ontario), and observed survival of unfrozen individuals exposed to -50°C for one hour. This cold tolerance was accompanied by higher hemolymph osmolality and glycerol concentration than in the SW Ontario individuals. To distinguish between phenotypic plasticity and local adaptation, in 2020/21 we overwintered Winnipeg-sourced individuals either outdoors in SW Ontario or in a simulated Winnipeg winter. Simulated Winnipeg winter individuals had cold tolerance similar to those overwintered in Winnipeg, while SW Ontario overwintered individuals had cold tolerance similar to those collected previously in the region. The simulated winter individuals had higher hemolymph glycerol concentrations than SW Ontario overwintered animals, at least in part due to greater dehydration. Thus, *A. planipennis* are cold-tolerant enough to survive some of the harshest winters where their host trees can grow, and most likely attain this cold tolerance via phenotypic plasticity. These findings raise the importance of delineating sensitivity of conclusions to unexpected phenotypic plasticity when predicting potential distributions of new invasives or responses to climate change.

## Highlights

• Emerald Ash Borer persist in Winnipeg, Manitoba, Canada, after polar vortex winters
• Winnipeg EAB are more cold-tolerant than their counterparts in Southern Ontario
• Winnipeg cold tolerance is accompanied by high hemolymph [glycerol] and osmolality
• Winnipeg cold tolerance results from phenotypic plasticity, not genetic adaptation

## Introduction

Invasive insects are economically and ecologically costly globally (Bradshaw et al., 2016). Predicting the potential distribution of potential, new, and range-expanding invasives is therefore key to risk assessment and management of invasive species (Srivastava et al., 2019). Distribution models can be either based on habitat characteristics inferred from the native range (Ecological Niche Models; Lobo, 2016), or based on ecological or physiological data (Mechanistic Models; Maino et al., 2016). Neither of these approaches consistently yields accurate predictions of potential distribution (Barbet-Massin et al., 2018, Lee-Yaw et al., 2021), particularly when the invaded environment is substantially different to environments found in the native range. One potential source of error in these models is a failure to account for selection on or plasticity of the physiology of invasive populations (Colautti and Lau, 2015, Lee-Yaw et al., 2021) and among-population variation in the physiological parameters that limit distribution (Sinclair et al., 2012). Here we show how unaccounted-for phenotypic plasticity could be responsible for the significant under-prediction of an invasive species’ potential geographic range.

The emerald ash borer *Agrilus planipennis* Fairmaire, 1888 (Coleoptera: Buprestidae) is a destructive invasive pest of ash (*Fraxinus*) in North America and Europe (Herms and McCullough, 2014). In southwestern Ontario, Canada (close to the North American introduction point), most *A. planipennis* overwinter as freeze-avoidant prepupae, accumulating up to 4 M glycerol in the hemolymph, and depressing the supercooling point (SCP, the temperature at which they freeze) to allow them to survive temperatures as low as -35°C (Crosthwaite et al., 2011). Based on this information, *A. planipennis* should survive winter across most of North America; however, modelling suggests that extreme winters in some Canadian cities should be too cold to allow *A. planipennis* to persist (Cuddington et al., 2018). In particular, so-called ‘polar vortex’ events, wherein cold Arctic air masses are pushed south, yield extremely low temperatures at temperate latitudes (Overland and Wang, 2019). These temperatures can be well below the lowest SCPs recorded for *A. planipennis* (MacQuarrie et al., 2019) and might be expected to extirpate local populations. Indeed, the 2016 polar vortex event appeared to kill a significant number of *A. planipennis* in New York state, USA (Jones et al., 2017). However, in November 2017 a population of *A. planipennis* was discovered in Winnipeg, Manitoba (CFIA, 2017), which should be outside the range of survivable winter conditions for this species.

Models predicting organisms’ geographic ranges seldom account for among-population variation in physiological parameters (Benito Garzón et al., 2019, Bush et al., 2016, Peterson et al., 2019), partly because the magnitude of this variation is often unknown. In the case of *A. planipennis*, Cuddington et al. (2018) and MacQuarrie et al. (2019) assume that cold tolerance is invariant across the geographic range – a reasonable assumption, given that the Ontario population appears to be derived from a single source (Keever et al., 2013), and host species has no apparent impact on cold tolerance (Christianson and Venette, 2018). However, cold tolerance can change through both physiological plasticity and natural selection, leading to significant among-population variation in overwintering capacity in some insects (Sinclair et al., 2012). For example, northern populations of the mosquito *Aedes albopictus* evolved increased diapause incidence in the span of c. 30 years in North America (Medley et al., 2019). Local adaptation (i.e. regional genetic adaptation) to regional climate appears to arise rapidly in invasive species, although the rate and likelihood of this adaptation is still being explored (Colautti et al., 2015). For example, the polar vortex event of early 2019 led to positive selection for cold tolerance in lizards in Florida, USA (Stroud et al., 2020). On the other hand, phenotypic plasticity can also be associated with successful invasions: the most-invasive *Impatiens* species in Europe is more physiologically plastic than its less-successful congeners (Peterson et al., 2019). Insect cold tolerance can be remarkably plastic. In SW Ontario, for example, overwintering *A. planipennis* decrease their SCP from c. -22°C in October to c. -30°C in February (Crosthwaite et al., 2011), and lose their cold tolerance after ten days above +10°C (Sobek-Swant et al., 2012). Thus, models based on data from a single location can misidentify temperature-based distribution limits if an invasive species evolves additional cold tolerance or has unrecognised capacity for phenotypic plasticity.

Based on winter temperatures and previous cold tolerance estimates Cuddington et al. (2018) had predicted a low probability of *A. planipennis* establishment in Winnipeg. Indeed, the discovery in 2017 was preceded by a polar vortex event in 2016 (Overland and Wang, 2016), and followed by a polar vortex in early 2019, with January air temperatures as low as -39.9°C recorded at one station in Winnipeg (Winnipeg A CS, accessible at https://climate.weather.gc.ca/climate_data/daily_data_e.html). These temperatures were below 95% of supercooling points reported by Crosthwaite et al. (2011), and would be expected to extirpate the population (Cuddington et al., 2018, MacQuarrie et al., 2019). However, *A. planipennis* persisted in Winnipeg after the polar vortex as evidenced by our ability to collect viable *A. planipennis* for the present study. Thus, it is likely that Winnipeg *A. planipennis* are more cold-tolerant than the southern counterparts with which they share a common introduction event (Herms et al., 2014). We can address the underlying cause of this improved cold tolerance with two competing hypotheses: first, Winnipeg *A. planipennis* have evolved increased cold tolerance. In this case, we would predict that Winnipeg-sourced *A. planipennis* would express increased cold tolerance regardless of their overwintering environment. Second, the increased cold tolerance of Winnipeg *A. planipennis* is a result of phenotypic plasticity. In this case, Winnipeg *A. planipennis* would exhibit increased cold tolerance only in response to appropriate environmental cues.

We addressed these hypotheses by comparing the cold tolerance of overwintering *A. planipennis* collected in Winnipeg and the southern part of the Ontario range in the winter of 2018-2019. Because *A. planipennis* has all but extirpated *Fraxinus* hosts from the SW Ontario sites studied by Crosthwaite et al. (2011), we collected animals from one of the most southerly large populations in Ontario (Barrie, ON), and wintered them outdoors in London, ON. To distinguish between plasticity and genetic adaptation, in 2020/2021 we overwintered Winnipeg-collected individuals either outdoors in London, ON, or in the laboratory under simulated Winnipeg autumn and early winter conditions. We found that Winnipeg-collected individuals were considerably more cold-tolerant than their southern counterparts, with some survival after exposure to -50°C. Winnipeg individuals overwintered in London, ON (even during a Polar Vortex event) were considerably less cold-tolerant than their counterparts overwintered in simulated Winnipeg conditions, from which we conclude that the increased cold tolerance in the Winnipeg population is a result of phenotypic plasticity. Thus, the potential range of *A. planipennis* is significantly broader than previously thought. Furthermore, we show that plasticity in cold tolerance can yield substantial physiological differences among populations of recently introduced species that is beyond the variance usually accounted for in distribution models used for risk assessment.

## Methods

**Figure 1:**
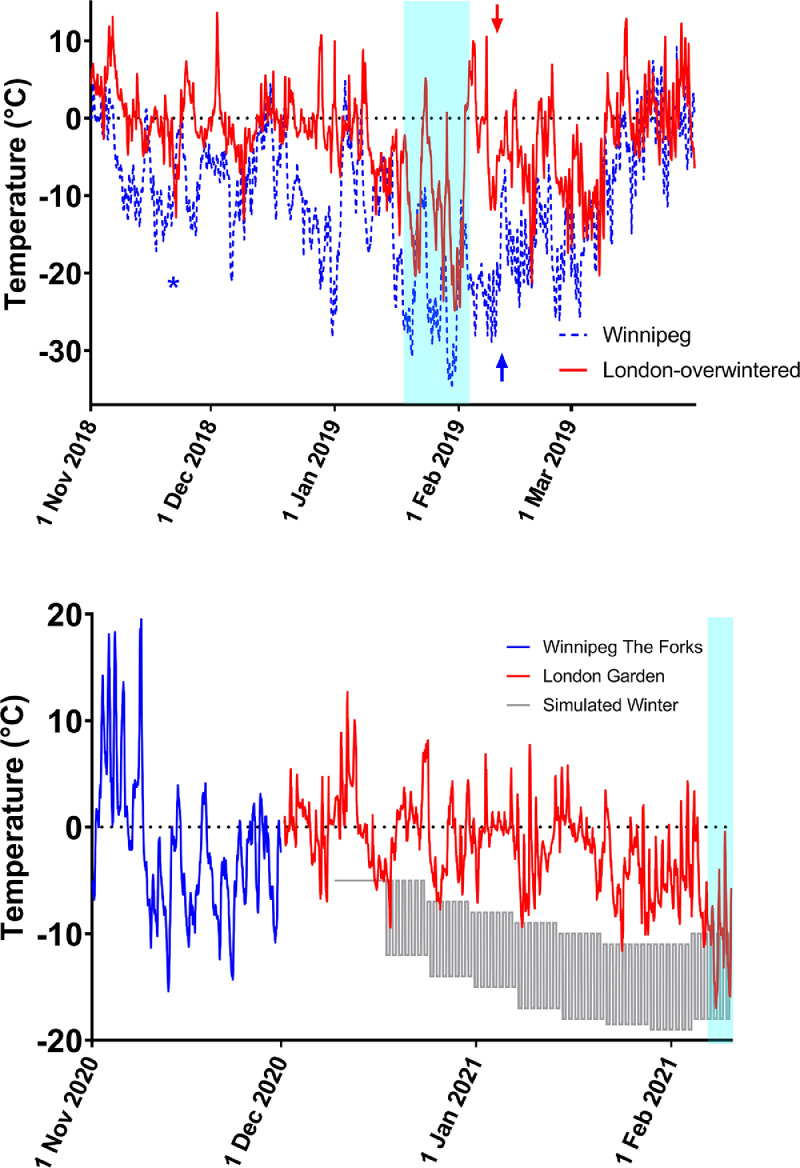
Winnipeg and London Environment Canada field temperature data in 2018-2019 (A), and field/simulated winter conditions for 2020-2021 (B). In (A), asterisk indicate the date infested trees were felled in Winnipeg (trees in Barrie, ON were felled 24 October, outside the range of the figure), arrows indicate the dates the infested bolts were removed from the field. Data are from Environment Canada stations ‘London Station A’ or ‘Winnipeg The Forks’. In (B), all animals experienced the Winnipeg autumn (Environment Canada ‘Winnipeg The Forks’ data; solid blue line) before being shipped to London, ON, where they experienced a London, ON winter (solid red line – temperature from a data logger placed near the storage location) or a laboratory simulation of a Winnipeg winter (blue dashed line); see text for details. Blue boxes indicate the range of the polar vortex events mentioned in the text.

For 2018/2019, we use hourly air temperatures recorded by Environment Canada (https://climate.weather.gc.ca/historical_data/search_historic_data_e.html) in Winnipeg (‘Winnipeg The Forks’ station) and London (‘London A’ station) to estimate the conditions experienced by our experimental animals (Figure 1A). For 2020/2021, we use a combination of Environment Canada Temperatures from Winnipeg Forks, temperatures from a thermistor probe (connected to a Hobo UI2 data logger, Onset Computer, Bourne, MA, USA) placed next to the logs in the London, ON, overwintering site, and the temperature settings of our experimental chamber (Figure 1B).

In SW Ontario and most of the USA, *A. planipennis* is univoltine, overwintering as a prepupa, pupating and emerging in the spring, and developing through four larval instars in the summer (Herms et al., 2014). In a few cases in these southern habitats (and commonly in more northern habitats), the life cycle becomes semivoltine, with larvae as well as prepupae overwintering (Jones et al., 2019, Orlova-Bienkowskaja and Bieńkowski, 2016). In prepupae, cold tolerance is rapidly acquired in the fall, with low SCPs accompanied by the accumulation of significant amounts of glycerol in the hemolymph (Crosthwaite et al., 2011). The cold tolerance of overwintering larvae has not been explored in depth.

To compare Winnipeg *A. planipennis* to their southern counterparts, we overwintered animals outdoors in either Winnipeg or London, ON, before measurement in the laboratory. We harvested eight *A. planipennis*-infested bolts (short lengths of tree stem or main branch) from *F. pennsylvanica* and *F. nigra* trees in Winnipeg, Manitoba (49.88°N, 97.12°W), on 21 November 2018. These bolts were kept outside at the source location until 31 January 2019 when they were shipped to the Great Lakes Forestry Centre in Sault Ste. Marie, Ontario. The bolts arrived on 1 February and where they were placed in a controlled environment chamber at -7°C in darkness until 11 February, at which point insects were extracted from the bolts, placed individually with moistened filter paper (remoistened weekly) in the wells of 12-well cell culture plates, and shipped to Western University where they arrived on February 13, 2019. These animals were kept in an incubator at 0°C in complete darkness and used in experiments within two weeks. For southern samples, we harvested 12 infested bolts from a woodlot near Barrie in Oro-Medonte Township, Ontario (44.5°N 79.5°W) on 24 October 2018, placed them in plastic bins and transferred them to London, ON (43.0°N 81.2°W) on 25 October, 2018. These bolts were stored outside in secure wire mesh-covered bins until 10 February 2019 (‘London-overwintered’) when we transferred them to a dark incubator at 0°C, and extracted the animals in a walk-in chamber at 4°C (these bolts were stored for up to two weeks and used simultaneously in experiments with the Winnipeg animals). These animals were placed on moistened filter paper in individual wells of 6-well cell culture plates and stored in a dark incubator at 0°C until use in experiments within 24 h.

In 2020/2021 we conducted a split-environment experiment to distinguish genetic adaptation from plasticity. Covid-19-related restrictions prevented us from collecting samples from Barrie or overwintering samples in Winnipeg (precluding a reciprocal transfer experiment). Instead, we overwintered Winnipeg-collected larvae outdoors in London, ON, or in a simulated Winnipeg winter in the laboratory before measurement. We harvested 16 *A. planipennis*-infested *F. pennsylvanica* bolts on 17 November 2020 and shipped them in closed containers directly to London, ON where they arrived on 19 November, and were stored in a common outdoor environment until 1 December 2020. We divided these bolts into two groups (n=8 bolts each). One group was overwintered outside in London, ON (as described above, called ‘Winnipeg-London winter’ hereafter) until 10 February, when they were transferred to a dark incubator at 0°C and extracted and treated as described above between 11 and 19 February 2021. The second group of bolts was kept outside in the London location until December 10. These bolts were transferred to a walk-in chamber at -5°C until 18 December, when they were transferred to the custom-built ‘Earth Biome’ in Western's Biotron (an LT-200 chamber controlled by v-Net software, Biochambers, Winnipeg, Manitoba) for a simulated winter (‘Winnipeg-simulated winter’). The simulated winter fluctuated between the average weekly daytime and nighttime temperatures for Winnipeg (Environment Canada Station “Winnipeg The Forks”) from 1 December to 31 January 2010-2020. We began the simulated winter on 10 December and proceeded until 11 February, after which the temperature cycled between -10°C and -18°C; see Figure 1B for temperature profile. After 11 February *A. planipennis* were extracted for use in experiments over a period of ten days.

We extracted *A. planipennis* larvae and prepupae from bolts at 4°C as previously described (Crosthwaite et al., 2011). We separated prepupae, which had a characteristic J-posture and were found in pupation chambers from larvae, which did not have the J-posture and were found in galleries (Herms et al., 2014). We confirmed their identity of a subset of individuals (n= 21 from the Winnipeg population and n=8 from Barrie in 2018/2019) by sequencing a fragment of the mitochondrial COI gene (i.e. DNA barcode; Hebert et al., 2003). Briefly, we amplified COI using primers LCO1490K and HCO2198K (Simon et al., 1994), purified the products according to the service provider's protocols and sent them for sequencing off site (Eurofins Genomics, Louisville, KY, USA). We compared the sequences to *A. planipennis* diagnostic sequences described by Kelnarova et al. (2018). Sequences are deposited in NCBI GenBank under accession numbers OM218599-OM218627.

### Cold tolerance

In 2018/2019, we measured SCPs in cohorts of 5-15 randomly-assigned individuals to yield a final n=20 individuals/developmental stage/population. Our sample sizes were more restricted in 2020/2021. In 2021, we measured SCPs of nine larvae and four prepupae from the London-overwintered group, and 26 larvae and six prepupae from the Winnipeg-simulated winter group. We measured SCPs using the general approach described by Crosthwaite et al. (2011); briefly, we placed individuals in contact with type-T thermocouples in 1.7 mL microcentrifuge tubes in a copper block cooled by a custom-built Peltier-effect cooling device (described by Anthony et al., 2019). We placed type-T thermocouples in contact with the animal and recorded the temperature using a PicoTech TC-08 interface and Picolog software (v 6.1; Pico Technology, Cambridge, UK). We cooled larvae and prepupae from +2°C to the SCP at 0.5°C/min and identified the SCP as the lowest temperature prior to the freezing exotherm.

In 2018/2019, we separately estimated the lower lethal temperature of larvae and prepupae from both populations using the general method described by Sinclair et al. (2015). Briefly, we placed individual *A. planipennis* into 1.7 mL tubes and cooled them as described above at 0.25°C/min from +2°C to -10, -25, -35, -45, -50, and -60°C where they remained for one hour (two groups of five animals per temperature/life stage/population combination – sample size was restricted by the availability of animals). We rewarmed individuals at 0.25°C/min to +20°C and placed them in six-well plates with moist filter paper at room temperature (approximately 20°C) before assessing survival after 24 h. We considered individuals that were visibly pumping hemolymph and that moved in response to light prodding with a fine paintbrush to be alive.

### Hemolymph composition

In 2018/19, we measured hemolymph osmolality in ten individuals/life stage/population; in 2021 we measured hemolymph osmolality in five larvae and three prepupae from the London-overwintered group and two larvae and four prepupae from the Winnipeg-simulated winter group. We measured hemolymph osmolality using a nanolitre osmometer (Otago Osmometers, Dunedin, New Zealand) as described previously by Crosthwaite et al. (2011). We diluted 2 µl of hemolymph in 98 µl of 0.05 % Tween 20 and further diluted in 0.05% Tween 20 to within the linear range of the glycerol assay. We quantified hemolymph glycerol in 30 µL samples of hemolymph after incubation with free glycerol reagent (Sigma Aldrich, Inc. St. Louis MO, USA), using absorbance at 540 nm as described previously (Crosthwaite et al., 2011).

### Data analysis and availability

We compared supercooling points, osmolality, mass, and glycerol content among populations (or winter environments) and life stages using a two-way ANOVA with Satterthwaite's correction for unequal variances (where necessary) and Tukey's HSD *post-hoc* tests using Prism v9.1.2 (GraphPad software, San Diego, CA, USA). We did not have sufficient individuals to perform formal statistical tests on the lower lethal temperature data. Mean ± standard error of the mean (SEM) are presented unless otherwise stated. Our data are available as an Excel spreadsheet in the supplementary material and on Dryad (https://doi.org/10.5061/dryad.1c59zw3wv).

## Results

In Winnipeg, Manitoba, Canada, the 2019 polar vortex event lasted 15 days [20 January – 4 February] during which the minimum air temperature at the Winnipeg Forks station was -36.8°C, and the maximum temperature was -9.0°C (Fig. 1A). Over the same period in London, the minimum air temperature was -24.8°C and the maximum temperature was -1.4°C (Fig 1A).

Larvae and prepupae from both populations of *A. planipennis* were freeze avoidant in both years: in all laboratory experiments, all individuals that froze died.

### Population comparison – Winnipeg vs. London-overwintered

**Figure 2:**
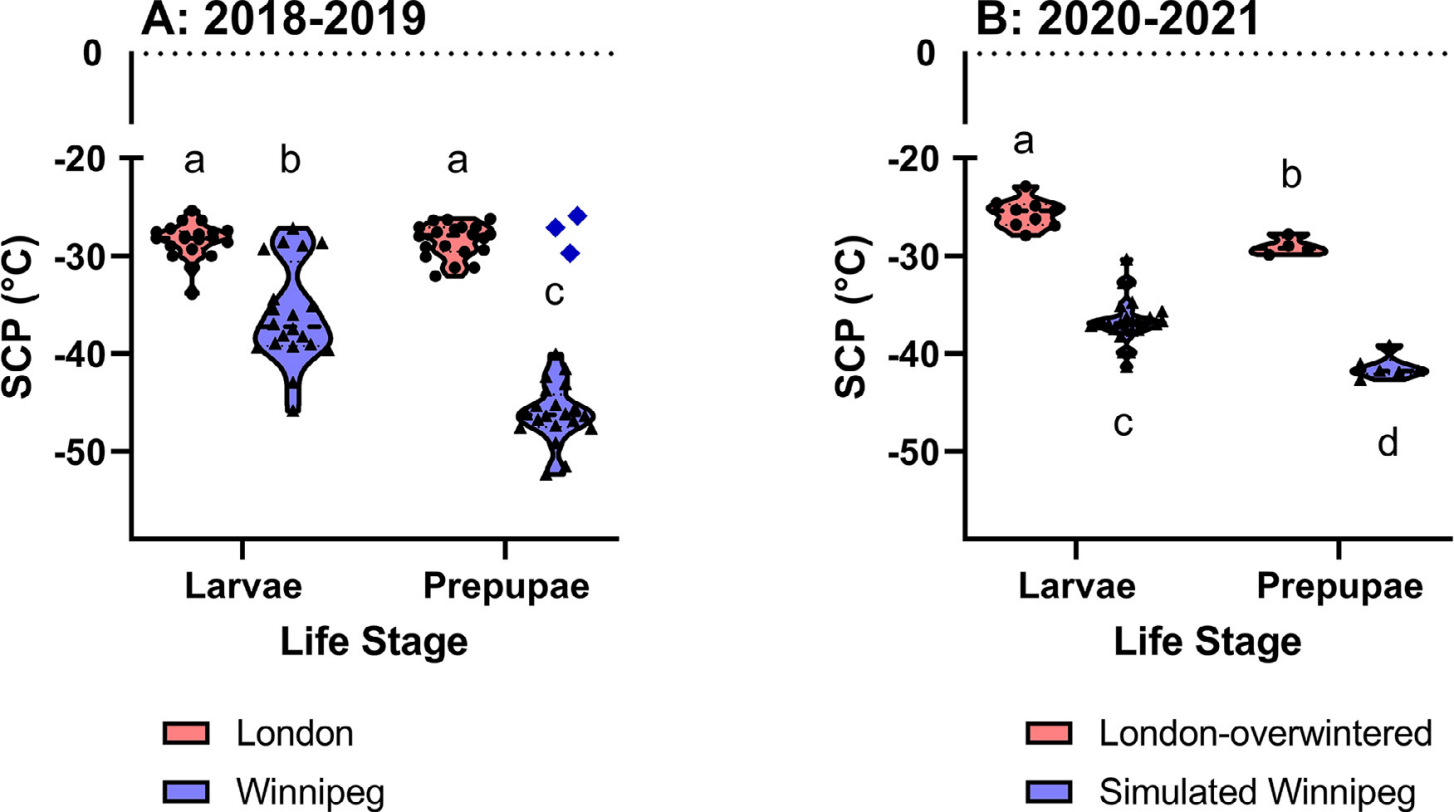
Supercooling points (SCP) of *A. planipennis* collected from Barrie, Ontario in 2018 and overwintered in London (London, A), collected in Winnipeg, Manitoba (2019, A), or collected in Winnipeg, Manitoba in 2020 and overwintered outdoors in London, ON or in a simulated Winnipeg winter (B). Groups with different letters within a panel have significantly different means (see Table 1 for statistics for panel A, and Table 2 for statistics for panel B). Small black symbols indicate individual data points, violin plots indicate distributions; the three outlier prepupae from Winnipeg in panel A were aberrant individuals, and were excluded from the analyses (see text for details).

Winnipeg *A. planipennis* were extremely cold-tolerant in 2019: the lowest SCP we measured in a prepupa was -52°C, and the lowest larval SCP was -45°C (Fig. 2A). By contrast, in 2019 the London-overwintered *A. planipennis* (which were sourced from the Barrie, ON population) were significantly less cold-tolerant than their Winnipeg counterparts (Fig 2A; Table 1), with minimum SCPs of -33°C and -32°C for prepupae and larvae, respectively. The SCPs of Winnipeg animals were more variable than those of prepupae, with four Winnipeg larvae and three Winnipeg prepupae having relatively high SCPs that were similar to those of London-overwintered larvae (Fig 2A). The larvae did not differ notably from the others in our sample in any way, so we included them in the analysis. However we excluded the three prepupae from our statistical analysis, because they were noticeably smaller than the other prepupae, and although they were in pupal chambers, were morphologically different to other prepupae (we speculate that they may be physiologically or developmentally unusual). Including these individuals in the analysis did not change the outcome of the statistical test or the conclusions (analysis not shown). The SCPs of London-overwintered larvae and prepupae did not differ significantly, whereas the SCPs of Winnipeg prepupae were significantly lower than those of Winnipeg larvae.

**Table 1:** Statistical comparisons of physiological parameters of *A. planipennis* larvae and prepupae (life stage) collected in Winnipeg, MB, and overwintered in or Barrie, ON (and overwintered in London, ON; Source) in the winter of 2018-2019. See Figures 3 and 4 for data presentation.

	F	df	*p*
**Supercooling points 2018-19**			
Life stage	**48.6**	**1,76**	**<0.001**
Source	**306**	**1,76**	**<0.001**
Source × life stage	**48.7**	**1,76**	**<0.001**
**Osmolality 2018-19**			
**Source**	**225**	**1,36**	**<0.001**
**Life Stage**	**11.6**	**1,36**	**0.002**
Source × life stage	0.381	1,36	0.541
**Glycerol 2018-19**			
**Life stage**	**3.42**	**1,36**	**0.073**
**Source**	**37.6**	**1,36**	**<0.001**
Source × life stage	3.02	1,36	0.091

**Figure 3:**
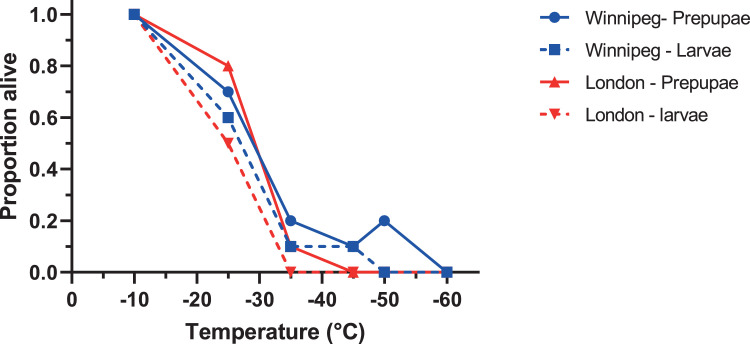
Survival of *Agrilus planipennis* larvae and prepupae after one hour exposure to low temperatures. Animals were collected in 2018 from Barrie, ON and overwintered in London, ON, or overwintered and collected in 2019 in Winnipeg, MB (see text for details). N= 10 individuals for each temperature × source × life stage combination.

In our lower lethal temperature experiment, Winnipeg *A. planipennis* with very low SCPs remained freeze avoidant, and we observed survival after exposure to very low temperatures. One larva from Winnipeg survived 1 h at -45°C while two Winnipeg prepupae survived 1 h at -50°C (Fig. 3). By contrast, the lowest temperature survived for one hour by larvae and prepupae overwintered in London-overwintered animals was -25°C and -35°C, respectively (Fig. 3). In these experiments, every individual that froze died, and every individual that was cold-exposed but did not freeze survived, irrespective of temperature.

**Figure 4:**
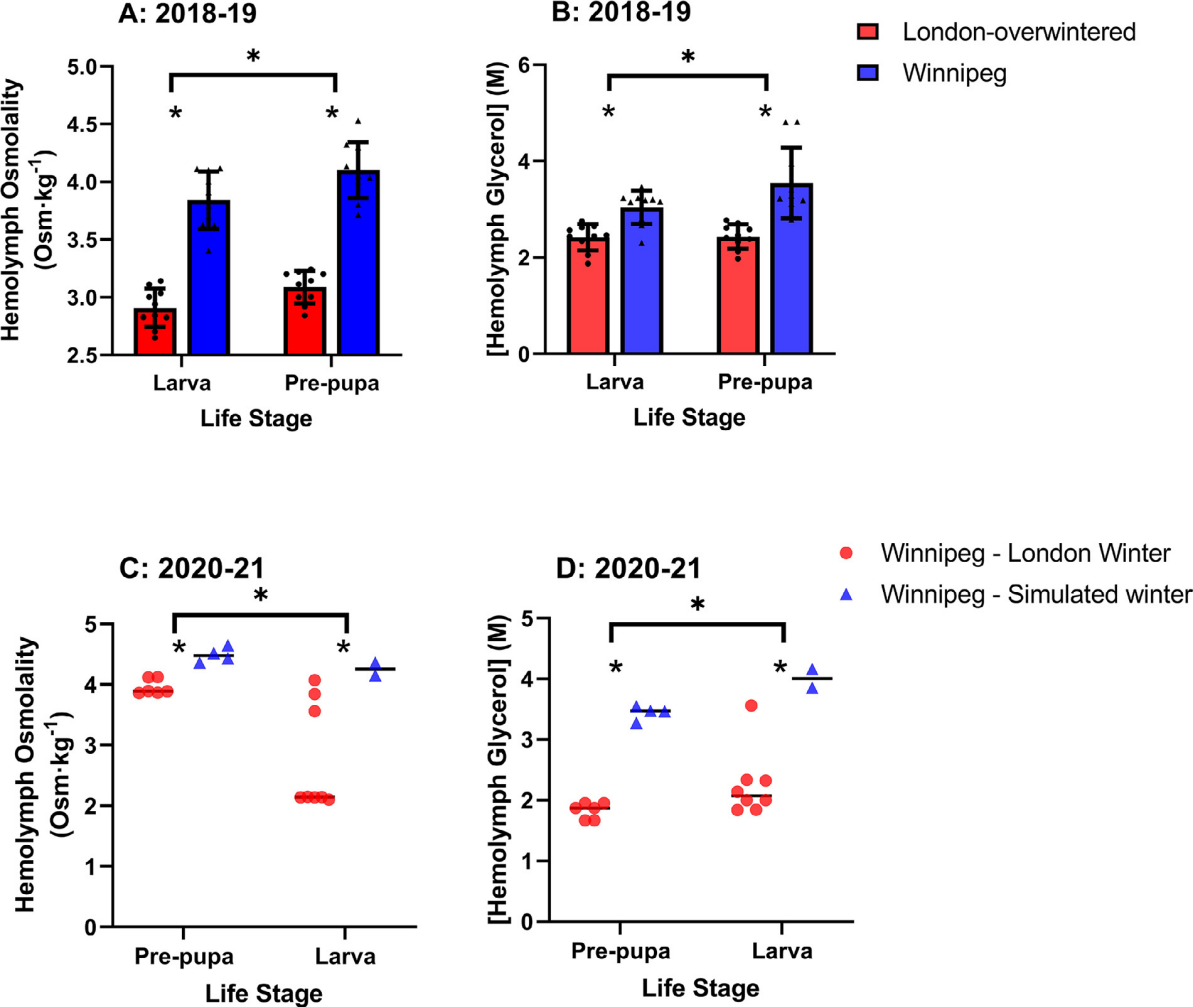
Hemolymph osmolality (A, C) and glycerol concentration (B, D) of overwintering *Agrilus planipennis* larvae and prepupae. In 2018-19 (A, B), animals were collected from Barrie, ON and overwintered in London, ON (London-overwintered), or overwintered and collected in Winnipeg, MB (see text for details). In 2020-21 (C, D), animals were all collected in Winnipeg, MB, and overwintered either outdoors in London, ON (Winnipeg – London Winter) or in a simulated Winnipeg winter in the laboratory (Winnipeg – Simulated winter); see text and Figure 1 for details. Individual symbols indicate individual data points; mean ± SEM presented for A and B, mean is indicated with a horizontal line in C and D. Asterisks indicate significant differences between source and life stage, there were no significant interaction effects (see Table 1 for statistics).

The variation in cold tolerance we observed in 2018-19 is reflected to some extent in variation in hemolymph composition. Winnipeg animals had hemolymph osmolality ranging from 3.4 Osm (in a larva) to 4.5 Osm (in a prepupa) whereas London-overwintered hemolymph osmolality ranged from 2.6 Osm (in a larva) to 3.2 Osm (in a prepupa; Fig 4A). Hemolymph osmolality was significantly higher in Winnipeg than London-overwintered animals for both life stages, and higher in prepupae than larvae; there was no source × life stage interaction, suggesting that this pattern is consistent among populations (Figure 4A; Table 1). These very high hemolymph osmolalities were accompanied by high hemolymph glycerol concentrations, up to 4.8 M in a prepupa from Winnipeg (Figure 4B). Hemolymph glycerol concentrations did not differ between life stages (and there was no source × life stage interaction), but hemolymph glycerol concentration was significantly higher in Winnipeg individuals than their London-overwintered counterparts (Figure 4B, Table 1).

### *Plasticity of cold tolerance in the Winnipeg* A. planipennis *population*

In 2020/2021, we overwintered Winnipeg-sourced individuals in London and simulated Winnipeg conditions. During 2020-2021, the London-overwintered (but Winnipeg-sourced) bolts experienced a brief polar vortex event from 6 February until the bolts were returned to the laboratory on Feb 10, during which the overnight temperature dropped below -15°C twice, with a minimum of -17°C (Fig 1B).

London-overwintered individuals had SCPs ranging from -22.9°C (a larva) to -29.9°C (in a prepupa), while Winnipeg-simulated winter individuals had significantly lower SCPs, ranging from -30.4 (in a larva) to -42.7°C (in a prepupa; Fig 2B). There was no winter environment × life stage interaction (Table 2). We had only a few measurements of hemolymph composition from this experiment (see Fig 4, Table 2). Hemolymph osmolality ranged from 2.1 Osm (in a London-overwintered larva) to 4.6 Osm (a Winnipeg-simulated winter prepupa), while glycerol concentrations ranged from 1.7 M (in a London-overwintered prepupa) to 4.2 M (in a Winnipeg-simulated winter larva). Hemolymph glycerol concentration and osmolality were both higher in prepupae than in larvae, and higher in Winnipeg-simulated winter animals than in London-overwintered animals but there was no winter environment × life stage interaction in either case.

**Table 2:** Statistical comparisons of physiological parameters of *A. planipennis* larvae and prepupae (life stage) collected and overwintered either outdoors in London, ON or in a simulated Winnipeg winter in the laboratory in the winter of 2020-21 (Source). See Figures 3 and 4 for data presentation.

	F	df	*p*
**Supercooling points 2020-21**			
**Life stage**	**31**	**1,41**	**<0.001**
**Winter environment**	**255**	**1,41**	**<0.001**
Winter environment × life stage	0.859	1,41	0.859
**Osmolality 2020-21**			
**Life stage**	**5.52**	**1,16**	**0.032**
**Winter environment**	**3.91**	**1,16**	**0.004**
Winter environment × life stage	2.49	1,16	0.134
**Glycerol 2020-21**			
**Life stage**	**6.35**	**1,16**	**0.022**
**Winter environment**	**72.9**	**1,16**	**<0.001**
Winter environment × life stage	0.132	1,16	0.072

## Discussion

Distribution models based on previous cold tolerance measurements from southwestern Ontario had suggested that the northerly distribution of *A. planipennis* in Canada would be limited by extremely cold winter conditions (Cuddington et al., 2018, MacQuarrie et al., 2019). However, such models assume that cold tolerance is invariant across the geographic range. Here we show that the persistence of *A. planipennis* in Winnipeg, Manitoba, after a severe polar vortex winter is because the SW Ontario measurements underestimated cold tolerance in the Winnipeg population. Further, we show that phenotypic plasticity underlies the difference in cold tolerance between the Winnipeg and SW Ontario populations.

Both the larvae and prepupae in our study were freeze avoidant, in keeping with previous observations for overwintering *A. planipennis* prepupae (Crosthwaite et al., 2011). The London-overwintered animals had SCPs similar to those described by Crosthwaite et al. (2011), in spite of being sourced from a population c. 160 km north of London, ON. However, the animals that had experienced a Winnipeg winter in 2018-19 had extremely low supercooling points – all of these (excluding the three unusual individuals) individuals had SCPs lower than any reported by Crosthwaite et al. (2011). Indeed, almost 75% of the Winnipeg-overwintered prepupal SCPs were below -45°C (Fig. 2A), placing them firmly among the lowest SCPs reported for hydrated insects (Lee, 2010, Sinclair, 1999). All larvae and prepupae in the simulated Winnipeg winter had lower SCPs than their London-overwintered counterparts sourced from the same location (Figure 2B). The mean SCPs of the simulated Winnipeg winter larvae and prepupae were 7 and 5°C higher (respectively) than those of the Winnipeg-overwintered individuals from 2018/19, suggesting that there may be yet further capacity for plasticity of SCP, perhaps elicited by the much lower temperatures the 2018/19 Winnipeg animals experienced in the field.

One possible explanation for the extreme cold tolerance in Winnipeg-overwintered (and -simulated winter) individuals is that selective mortality in the Winnipeg winter means that the surviving individuals included in our measurements represent only the lower range of the SCP distribution. However, none of the London-overwintered animals had SCPs approaching those in the Winnipeg-overwintered and simulated Winnipeg groups, suggesting that the two groups do not come from the same distributions. Furthermore, during collection we noted that survival in bolts that experienced a Winnipeg winter (simulated or actual) was similar or greater than mortality in those that experienced a London winter (see Supplementary Figure S1). Finally, the lowest temperature that the Winnipeg-simulated winter individuals were exposed to was -18°C, which would not be sufficient to modify the overall SCP distribution in the London-overwintered groups (which had no SCPs above -20°C). Thus, the low SCPs we observed in Winnipeg-overwintered and Winnipeg-simulated winter *A. planipennis* do not appear to be because we included only individuals that had already survived extreme cold exposure.

Overwintering *A. planipennis* had very high hemolymph osmolality which appears to be driven by high hemolymph glycerol concentrations. The glycerol concentrations we observed in the Winnipeg animals were higher than those reported by Crosthwaite et al. (2011), but by a magnitude roughly consistent with the lower SCPs we observed. The high hemolymph osmolality we observed cannot be explained entirely by glycerol; it is possible that these animals accumulate other low molecular weight cryoprotectants [although Crosthwaite et al. (Crosthwaite et al., 2011) did not identify any other carbohydrate or polyol cryoprotectants, they did not screen for other types of cryoprotectant molecule, such as the free amino acid proline (Koštál et al., 2011)]. Dehydration over winter is another possible explanation for low supercooling points and high hemolymph concentrations (Danks, 2000, Sformo et al., 2010, Zachariassen, 1991). In London, the water content of mid-winter pre-pupae was only c. 6% lower than at the start of winter (Crosthwaite et al., 2011). By contrast, water content was c. 42 % and 23 % lower in simulated-winter compared to London-overwintered larvae and prepupae, respectively (Fig. S2). Thus, water loss likely explains much of the increased hemolymph concentrations and improved cold tolerance we observed, although it is beyond the scope of this study to explore this mechanism further.

The hemolymph of overwintering *A. planipennis* (especially those from Winnipeg) was highly viscous, bordering on gelatinous (MED, personal observation), making it difficult to detect thermal hysteresis during osmometry. Crosthwaite et al. (2011) reported a small amount of thermal hysteresis activity in overwintering *A. planipennis* prepupal hemolymph, and we did observe hints of thermal hysteresis in both larvae and prepupae from both populations (MED, personal observation). We hypothesise that these low levels of thermal hysteresis likely stabilize highly supercooled fluids (cf. Zachariassen and Husby, 1982), facilitating survival of animals supercooled to these very low temperatures.

At latitudes with short growing seasons, *A. planipennis* overwinter as larvae and extend their development into a semivoltine life cycle (Poland et al., 2015). Larval cold tolerance has not been well-explored in this species, but the few (n=18) larval SCPs measured during Crosthwaite et al.’s (2011) study in London did not differ from the SCPs of prepupae (J.C. Crosthwaite and BJS, unpublished data). Although our data from the Barrie population support this, we found that Winnipeg larvae were less cold-tolerant (i.e. had higher SCPs) than prepupae (Fig 2A), and that this difference may be driven by reduced plasticity in larvae compared to prepupae (Fig 2B; we did not have sufficient data to compare among larval instars). Among-life stage variation in plasticity is seldom accounted for in distribution models, but our observations suggest that among-stage variation in plasticity could affect the ability of an invasive species to adopt a multi-year life cycle.

We identified two hypotheses for the increased cold tolerance in the Winnipeg *A. planipennis* population. First, we hypothesized that the Winnipeg population has evolved increased cold tolerance. Second, we hypothesized that *A. planipennis* is phenotypically plastic and the Winnipeg population has acclimatized to very cold autumn and winter temperatures. Although Covid-19 restrictions prevented us from carrying out a reciprocal transplant experiment, we did use a split-environment design, overwintering Winnipeg-sourced individuals in London conditions and in a simulated Winnipeg winter. This revealed striking phenotypic plasticity: supercooling points of London-overwintered individuals were similar to those described previously for London *A. planipennis* (Crosthwaite et al., 2011), whereas the simulated Winnipeg winter yielded SCPs almost as low as those we found for individuals that experienced the Winnipeg polar vortex in 2019, and more than 10°C below the lowest SCPs Crosthwaite et al. (2011) recorded for any individual in southwestern Ontario. Thus, we conclude that the extreme cold tolerance we report in the Winnipeg population is most likely due to extreme plasticity of cold tolerance in the *A. planipennis* population established there.

Although there are not yet any whole-genome sequence comparisons, the *A. planipennis* populations in Ontario appear to be genetically homogeneous (Keever et al., 2013) and have reduced diversity relative to Asian source populations (Bray et al., 2011). Quebec populations (established c. 2008) are somewhat differentiated from the Ontario/Michigan genotype (Keever et al. 2013); but the population from Sault Ste. Marie, ON, the most northerly population in 2013 (and the suspected source of the Winnipeg population) was not distinct from the rest of Ontario (Keever et al., 2013). Indeed, all the COI barcodes from individuals included in our study were identical. This genetic homogeneity (i.e. a lack of variation for selection to act on), is coupled with a lack of time to accrue new variation (the Winnipeg population probably dates to between 2011 and 2014; CJKM Pers. Obs.). Combined with the close similarity of the cold tolerance of London-reared Winnipeg individuals with the cold tolerance of individuals from Barrie, ON [or indeed London, ON (Crosthwaite et al., 2011)] we believe that plasticity is a more likely explanation than evolution as the cause of the extreme cold tolerance in this case.

While we think it is unlikely, our data do not allow us to rule out among-population variation in cold tolerance plasticity. There are few explorations of among -population variation in plasticity of overwintering biology [but see (Williams et al., 2012) for an example with metabolic suppression]. Among freeze-avoidant species, larvae of the spruce budworm *Choristoneura fumiferana* from sub-Arctic Inuvik can suppress their SCPs from an average of c. -32°C to an average of c. -37°C, whereas their counterparts from more southerly New Brunswick or Quebec in Eastern Canada maintained a steady SCP of c. -32°C after the same treatment (Butterson et al., 2021). We note that the magnitude of SCP plasticity in *C. fumiferana* (c. 5°C) is considerably less than the c. 12°C SCP depression we induced in *A. planipennis*, and that these *C. fumiferana* populations have been geographically separated and present in their current locations for thousands of years (Lumley et al., 2020). We do not have plasticity information for our Barrie population, although we note that overwintering these animals in London, ON (a milder climate than Barrie, ON) yielded similar cold tolerance to what we saw in the (former) SW Ontario populations (Crosthwaite et al., 2011), suggesting that a similar plasticity exists in that population. However, we cannot speculate about whether all North American *A. planipennis* populations have capacity for such extreme phenotypic plasticity, although in future it will be interesting to measure cold tolerance plasticity in the *A. planipennis* populations expanding into mild habitats in the USA and Europe (Nalepa et al., 2021, Volkovitsh et al., 2021).

Most previous studies of plasticity of insect cold tolerance in freeze-avoidant species have compared non-winter physiology to winter physiology (e.g. Krunic and Salt, 1970), made comparisons of field-collected individuals within winter (e.g. Rickards et al., 1987), or have explored deacclimation of animals removed from winter conditions (e.g. Sobek-Swant et al., 2012). These studies are the foundation for most of our correlation-based understanding of the mechanisms underlying insect cold tolerance (Lee, 2010), but provide little insight into the limits of cold tolerance. Laboratory studies of cold tolerance plasticity in freeze-avoidant insects appear rare [see Butterson et al. (2021) for a recent example]. To our knowledge, the c. 12°C difference in mean SCP we were able to induce in overwintering *A. planipennis* is the highest reported for plasticity of freeze-avoidant insects already acclimatized to their overwintering state.

In summary, we have shown that *A. planipennis* can be significantly more cold tolerant than revealed by previous single-locality studies. This increased cold tolerance is sufficient to allow them to survive unusually extreme winter events in a location that previous models suggest should have a low likelihood of establishment due to winter conditions. Furthermore, this extreme cold tolerance is probably a result of phenotypic plasticity, rather than genetic adaptation, which means that variation in cold tolerance among populations can manifest in the first winter after an introduction event, rather than taking time for natural selection to take its course.

We conclude that mechanistic species distribution models based on physiological tolerances from laboratory conditions or a single field population, should include sensitivity analyses to identify the impact of plasticity on their conclusions (and perhaps delimit the magnitude of plasticity that would be necessary to substantially change predicted distributions). Although few mechanistic models thus far have incorporated plasticity, they are clearly well-suited to this approach (Maino et al., 2016). It could be argued that bioclimatic envelope models, by contrast, already incorporate plasticity in their parameterization of native range distributions (Lobo, 2016). However, such models often lack data from native range edges, assume that distribution limits are set by abiotic conditions in the native range, and assume that the parameter space that elicits plasticity in the introduced range exists in the native range. Thus, plasticity in the introduced range could still yield novel phenotypes, even in species well-known in their native range. Finally, in the context of climate change, our data contribute to the emerging picture that suggests that phenotypic plasticity is extremely important in allowing a species (invasive or otherwise) to expand its geographic distribution (see also Gvozdik, 2012, Huey et al., 2012, Rodrigues and Beldade, 2020, Sgró et al., 2016, Somero, 2010).

## CRediT authorship contribution statement

**Meghan E. Duell:** Investigation, Formal analysis, Data curation, Writing – original draft, Project administration. **Meghan T. Gray:** Investigation, Resources, Writing – review & editing. **Amanda D. Roe:** Conceptualization, Investigation, Writing – review & editing, Funding acquisition. **Chris J.K. MacQuarrie:** Conceptualization, Resources, Writing – review & editing, Funding acquisition. **Brent J. Sinclair:** Conceptualization, Formal analysis, Data curation, Writing – original draft, Visualization, Funding acquisition.

## Declaration of Competing Interest

The authors declare no financial conflicts associated with this Research. Brent J. Sinclair is Editor-in-Chief of *Current Research in Insect Science*, and Amanda D. Roe is an Editorial Board member of the journal. Given their roles, neither had any involvement in the evaluation or peer review of this manuscript, and neither have access to information regarding its peer review.

## Appendix Supplementary materials

Image, application 1

Image, application 2
