# Supplementary material for: Plasticity drives extreme cold tolerance of emerald ash borer (Agrilus planipennis) during a polar vortex
Source: Curr Res Insect Sci. 2022 Feb 3;2:100031. doi: 10.1016/j.cris.2022.100031 (PMC9387492; doi:10.1016/j.cris.2022.100031)
Supplement: Supplementary file 1 [file mmc1.docx]

Supplementary information for:

**Plasticity drives extreme cold tolerance of emerald ash borer (*Agrilus planipennis*) during a polar vortex**

Meghan E. Duell^1^, Meghan T. Gray^2^, Amanda D. Roe^2^, Chris J. K. MacQuarrie^2^, Brent J. Sinclair*^1^

^1^ Department of Biology, University of Western Ontario, London ON N6A 5B7

^2^ Great Lakes Forestry Centre, Sault Ste. Marie, ON, P6A 2E5

*Correspondence: Brent Sinclair, Department of Biology, Western University, London, ON, N6A 5B7, Canada; [bsincla7@uwo.ca](mailto:bsincla7@uwo.ca)

Figure S1

**Figure S1:** Survival of *A. planipennis* extracted from *Fraxinus* sp. bolts. London-overwintered bolts were collected in Barrie, ON, and overwintered in London, ON; Winnipeg (2018-19) bolts were collected from Winnipeg, and overwintered there. In 2020-21, infested bolts were collected from Winnipeg, MB, and overwintered either outdoors in London, ON [Winnipeg (London Winter)], or in a simulated winter in the laboratory [Winnipeg (simulated winter)]. Each data point indicates percent survival of all individuals in a bolt; total number of individuals in a bolt ranged from 2 to 52 (see supplementary spreadsheet for data). Parasitoids were only present in the London-overwintered (2018-19) bolts, accounting for high mortality of both larvae and prepupae in that group.

Figure S2

**Figure S2**: Water content of *A. planipennis* at the end of 2020-2021 winter. *Fraxinus* bolts were harvested in Winnipeg, MB, and either overwintered outdoors in London, ON, or in a simulated Winnipeg winter environment (see text for details). Water content was measured gravimetrically. Note log scale on Y-axis, because of the large range of sizes of larvae. Although mass-specific data are presented here for convenience, water contents were compared using an ANCOVA with dry mass as a covariate. Water content was significantly lower in prepupae compared to larvae (F_1,39_=15.567, p<0.001), and in simulated winter animals compared to London-overwintered animals (F_1,39_=9.746, p=0.003). The source × life stage interaction was not statistically significant (F_1,39_=1.625, p=0.210). Asterisks indicate significant differences among the groups from a Tukey’s *post hoc* test.
